# Robust Sparse Bayesian Learning-Based Off-Grid DOA Estimation Method for Vehicle Localization

**DOI:** 10.3390/s20010302

**Published:** 2020-01-05

**Authors:** Yun Ling, Huotao Gao, Sang Zhou, Lijuan Yang, Fangyu Ren

**Affiliations:** Electronic Information School, Wuhan University, Wuhan 430072, Chinazhousang@whu.edu.cn (S.Z.); yanglijuan@whu.edu.cn (L.Y.); renfangyu921@whu.com (F.R.)

**Keywords:** vehicle localization, passive bistatic radar, DOA estimation, sparse Bayesian learning, off-grid gap

## Abstract

With the rapid development of the Internet of Things (IoT), autonomous vehicles have been receiving more and more attention because they own many advantages compared with traditional vehicles. A robust and accurate vehicle localization system is critical to the safety and the efficiency of autonomous vehicles. The global positioning system (GPS) has been widely applied to the vehicle localization systems. However, the accuracy and the reliability of GPS have suffered in some scenarios. In this paper, we present a robust and accurate vehicle localization system consisting of a bistatic passive radar, in which the performance of localization is solely dependent on the accuracy of the proposed off-grid direction of arrival (DOA) estimation algorithm. Under the framework of sparse Bayesian learning (SBL), the source powers and the noise variance are estimated by a fast evidence maximization method, and the off-grid gap is effectively handled by an advanced grid refining strategy. Simulation results show that the proposed method exhibits better performance than the existing sparse signal representation-based algorithms, and performs well in the vehicle localization system.

## 1. Introduction

The emergence of the Internet of Things (IoT) and its applications have greatly affected the society and improved the quality of life [1,2]. For example, the IoT has been widely used in the autonomous vehicles [3,4], which are viewed as a potential solution to some critical problems, such as pollution, traffic congestion, and road accidents [5]. Almost all autonomous vehicles have five basic functions, including localization, perception, control, planning, and system management [6]. In order to implement correct driving decisions and actions, accurate knowledge of the vehicle’s location is required for the perception, planning, and control functional systems. Therefore, vehicle localization technology is critically important for autonomous vehicles. As the global positioning system (GPS) can offer a cheap and easily accessible solution for global localization, it is the first choice for vehicle localization. However, the accuracy and availability of the GPS signal cannot always meet the requirements of autonomous vehicle localization, due to satellite visibility interruption and signal multipath. To address this issue, many vehicle localization systems based on robust location algorithms with advanced sensors, such as RADAR [7], LiDAR [8,9], and camera [10], have been invented and become the focus of research.

In general, vehicle localization systems can work based on four principles, namely, time of arrival (TOA), time difference of arrival (TDOA), radio signal strength (RSS), and direction of arrival (DOA). In TOA approaches [11,12], a relative position to the reference point is obtained based on the time from a signal being sent to the reference point to the response signal back at the vehicle. The accuracy of the TOA approaches is susceptive to a synchronous clock. As a result, the perfect synchronization is hard to realize in practice. For TDOA approaches [13,14], the relative position of the vehicle can be calculated based on the time difference of signal arrivals. However, the accuracy of TDOA methods is limited due to their high sensitivity to measurements of time difference. In RSS methods [15,16], the attenuation of signal strength is measured to estimate the travel distance of the signal. However, the spatial fading characteristic of the signal is firstly required. In DOA approaches [17,18,19], the angle of the signal is measured via antenna arrays and the position can be calculated by the angle information. The accuracy of DOA approaches is only dependent on the accuracy of the angle estimation, which can be easily realized by the existing high accurate DOA estimation algorithms. DOA is thus the most efficient one among the four approaches.

Various DOA estimation methods have emerged during the past decades, among which subspace-based algorithms are best known with a wide range of applications [20]. However, they suffer from certain well-known constraints: prior knowledge of source number, uncorrelated circumstance, and a sufficient number of snapshots are required. The rapid development of sparse representation and its recent application in various fields [21,22,23,24,25,26,27,28,29,30,31,32] has offered a new perspective for DOA estimation. A great number of sparse representation-based DOA methods have emerged, which can be applied in several demanding scenarios that the subspace-based methods cannot. The recent DOA estimation based on sparse representation can be summarized into three categories. The first category concerns on-grid methods, including $l_{1}$-SVD [33] , SPICE [34], SBL [35], and MSBL [36]. For these kinds of methods, true DOAs are assumed to lie on a set of fixed grid points, therefore the existing sparse representation techniques can be applied directly. However, the gap between true DOA and its nearest grid point always exists in practice. The other two categories, namely, gridless methods and off-grid methods, can eliminate or narrow this gap. Gridless methods [37,38,39,40,41,42] operate in the continuous domain directly so that they can avoid the grid mismatch problem. Gridless methods have strong theoretical guarantees and can only be applied to the uniform or sparse linear arrays.

For the off-grid methods, a sampling grid is still required but the true DOAs are not restricted to be on the grid [43]. Han et al. (2015) [44] introduce a sparse total least-squares (STLS) approach, which can yield a maximum a posteriori (MAP) optimal estimate if the matrix perturbation caused by the basis mismatch is Gaussian. Off-grid sparse Bayesian inference (OGSBI) [45] takes a Bayesian perspective on the off-grid methods. The model of OGSBI is based on a first-order Taylor series expansion, and a Laplace prior is assumed to exploit the spatial sparsity of signals. Wu et al. (2016) [46] present an off-grid model based on a perturbed sparse Bayesian learning, in which a linear interpolation between two adjacent grid points is adopted. The off-grid methods mentioned above are still limited in such a way: On the one hand, a denser grid set is required for higher accuracy, which, however, may cause massive computation. On the other hand, a coarser grid can greatly reduce the computational workload but may introduce larger model errors. In order to address this issue, Dai et al. (2017) [47] give a root SBL (RSBL) method, which decreases computational workload by using a root method. It also maintains high accuracy with a coarse grid. However, RSBL is still quite time consuming at other steps in the iterative updating. In GRDOA [48], the grid points get reconfigurated via a grid refining and fission strategy, which makes the method more efficient and accurate than RSBL.

Vehicle localization based on DOA estimation is suitable and efficient in practice [17,18,49]. Since many recent DOA estimation algorithms are very fast, the moving distance of a vehicle during algorithms execution can be ignored. In other words, the moving vehicle can be assumed to be motionless in DOA estimation-based vehicle localization. The location of the vehicle can be obtained by the cross localization which merely depends on the accuracy of DOA estimation. Therefore, DOA estimation provides a suitable and efficient solution to the localization of a moving vehicle. For example, [17] provides a vehicle localization model based on two cooperating Multi-input multi-output (MIMO) radars and a cross localization method with a real-valued covariance vector-based SBL DOA estimation algorithm. In [18], three collaborative MIMO radars are used for vehicle localization. A robust SBL based DOA estimation approach is proposed to address the non-uniform noise as well as the off-grid issue. The location of the vehicle can be obtained by averaging the cross-localization results from each pair of the three MIMO radars.

The radar-based vehicle localization systems mentioned above [17,18,49] all use the active radars. In recent years, the passive radar systems have been considered to be promising due to their advantages over active radars, such as low-cost surveillance, reduced pollution of the electromagnetic environment, as well as covert operation [50,51,52,53,54]. The DOA estimation for the bistatic passive radar is investigated in [50], where a joint sparse Bayesian model is established to combine the measurements from both stations under the framework of SBL. Colone et al. (2012) [51] analyze the practical feasibility of a WiFi transmissions-based passive bistatic radar and demonstrates the potential of passive bistatic radar for local area surveillance applications, where vehicles and people can be detected and tracked. More ways of detecting and tracking vehicular and human targets based on passive bistatic radar systems can be found in [52,53,54]. However, to our best knowledge, vehicle localization systems using passive bistatic radar with SBL-based DOA estimation have rarely been studied in the existing literature, despite their high usefulness in practice.

A vehicle localization system based on a passive bistatic radar is low cost, low power usage, and reduced pollution of the electromagnetic environment, since passive bistatic radars have no transmitting stations and only receive data from existing source emitters [51]. The system is also robust as it is not affected by the weather and light. In addition, many existing SBL based DOA estimation methods can achieve higher accuracy than conventional ones [45,46,47,48]; the SBL-based DOA method is thus an efficient scheme for vehicle localization, as the localization method is merely dependent on the accuracy of DOA estimation. Therefore, it is worthwhile to investigate the vehicle localization systems using passive bistatic radar with an advanced SBL based DOA estimation algorithm.

In general, the main novelties and contributions of this paper can be summarized as follows:

(1) We provide a vehicle localization system consisting of a passive bistatic radar and the vehicle localization method via DOA estimation. The proposed system and method is practically feasible and valuable since it can achieve high efficiency, great robustness, low cost, as well as reduced electromagnetic pollution.
(2) Under the framework of SBL, we propose a novel off-grid DOA estimation approach. We introduce a fast evidence maximization method to update the hyperparameters to the off-grid DOA estimation domain, which can achieve faster convergence than the expectation maximization method used in many other SBL-based off-grid DOA approaches.
(3) Based on the conception of grid refining, we propose a new grid refining strategy consisting of a root process and a step process, which effectively reduces the off-grid errors. The grid refining strategy is less sensitive to the location of grid points and DOAs, which makes the proposed method more accurate than other DOA methods. The improvement is strengthened in good conditions with a large number of snapshots and high SNR. Furthermore, the grid refining strategy greatly speeds up the procedure of grid refining, making the proposed method more effective than other DOA methods.
(4) We conduct adequate simulations to verify the superiority and effectiveness of the proposed method. Then, the influence of the parameter $\theta_{step}$, the number of active grid points, and the different conditions on DOA estimation are examined. Then, we compare the proposed DOA method with GRDOA. Finally, a vehicle localization simulation is conducted to verify the feasibility of the proposed vehicle localization method.

## 2. Signal Model

### 2.1. System Model

**Figure 1:**
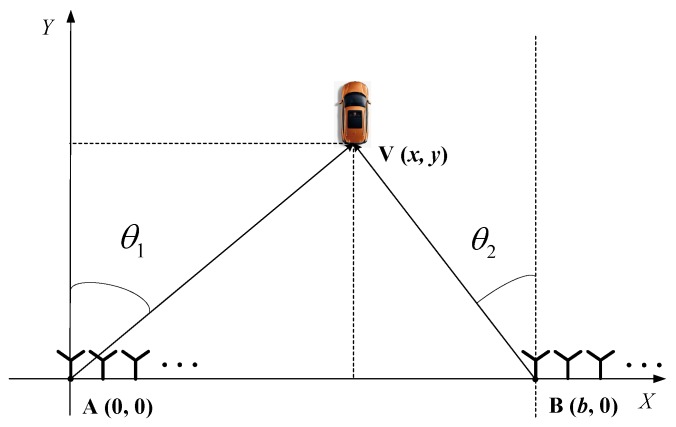
The DOA estimation-based vehicle localization model with passive bistatic radar.

Consider a vehicle localization system with a passive bistatic radar consisting of two stations that only receive data from source emitters, as shown in Figure 1. Point A and Point B are assumed to be the two receiving stations of the passive bistatic radar, which are placed on the same plane. The localization of a vehicle can be calculated via two DOAs, which can be estimated by these two stations, respectively. As shown in Figure 1, the location of one station is set as the reference point A(0,0) and another station is set as B(b,0). V(x,y) is the coordinate of the target vehicle, and its azimuth angles related to A and B are set as $\theta_{1}$ and $\theta_{2}$, respectively, which can be obtained via a DOA estimation method. It is easy to know that

(1) $$\tan\theta_{1}=\frac{x}{y},$$

(2) $$\tan\theta_{2}=\frac{b-x}{y},$$

and, based on Equations (1) and (2), the location of the target vehicle can be calculated as

(3) $$x=\frac{b\tan\theta_{1}}{\tan\theta_{1}+\tan\theta_{2}},$$

(4) $$y=\frac{b}{\tan\theta_{1}+\tan\theta_{2}}.$$

In the vehicle localization system, two stations are assumed to be configured and work in the same way, meaning that only one station needs to be described for DOA estimation because the other one works in the same way. Since the proposed DOA method is very fast, the moving distance of the vehicle during the method execution can be ignored. Namely, the moving vehicle can be assumed to be motionless.

### 2.2. Data Model

A uniform linear array (ULA) with *M* sensors and half-wavelength spacing is considered in each station of the vehicle localization system. Assume that there are *K* target vehicles in the same plane range, $\theta_{k}$ represents the DOA of the *k*th target where k=1,2,…,K, with K<M. In a passive radar system, the illuminating signal is emitted by a transmitter that is independent of the radar itself. The echo signal reflected by the target vehicles is considered as narrow-band and far-field. The signal received at the output of the array can be written as

(5) $$y\left(t\right)=As\left(t\right)+e\left(t\right),t\in \left(t_{1},t_{2},\ldots ,t_{L}\right),$$

where $y\left(t\right)={\left[y_{1}\left(t\right),y_{2}\left(t\right),\ldots ,y_{M}\left(t\right)\right]}^{T}$, $s\left(t\right)={\left[s_{1}\left(t\right),s_{2}\left(t\right),\ldots ,s_{K}\left(t\right)\right]}^{T}$, ${\left(\cdot \right)}^{T}$ is the transpose, *L* is the number of snapshots, $A=[a\left(\theta_{1}\right),a\left(\theta_{2}\right),\ldots ,a\left(\theta_{K}\right)]$ is an M×K matrix of steering vectors with $a\left(\theta_{k}\right)=\left[1,v_{\theta_{k}},\ldots ,v_{\theta_{k}}^{M-1}\right){]}^{T}$, $v_{\theta_{k}}=e^{-j2\pi d/\lambda \sin(\theta_{k})}$, *d* is the distance between adjacent sensors, and λ is the wavelength of the source. $e\left(t\right)={\left[e_{1}\left(t\right),e_{2}\left(t\right),\ldots ,e_{M}\left(t\right)\right]}^{T}$ is an unknown noise vector. The signals and the noise are assumed to be zero-mean, stationary complex Gaussian random processes and independent of each other. Furthermore, the noise is considered to be uncorrelated from sensor-to-sensor with common variance $\sigma^{2}$. Equation (5) can be compactly written as

(6) Y=AS+E,

with the definitions of $Y=[y\left(t_{1}\right),y\left(t_{2}\right),\ldots ,y\left(t_{L}\right)]$, $S=[s\left(t_{1}\right),s\left(t_{2}\right),\ldots ,s\left(t_{L}\right)]$, and $E=[e\left(t_{1}\right),e\left(t_{2}\right),\ldots ,e\left(t_{L}\right)]$.

To cast the DOA estimation as a sparse representation problem, the sparse signal model is constructed. Uniform sampling over DOA range is used in the conventional off-grid methods. Let $\tilde{\theta }=\left[{\tilde{\theta }}_{1},{\tilde{\theta }}_{2},\ldots ,{\tilde{\theta }}_{N}\right]$ be a fixed sampling grid in the range $\left[-\frac{\pi }{2},\frac{\pi }{2}\right]$, where *N* denotes the grid number (N≫M) and $r=\left|{\tilde{\theta }}_{2}-{\tilde{\theta }}_{1}\right|$ denotes the grid interval. If the grid is fine enough, the true DOAs will lie on (or, practically, close to) the grid. An M×N overcomplete matrix $A=[a\left({\tilde{\theta }}_{1}\right),a\left({\tilde{\theta }}_{2}\right),\ldots ,a\left({\tilde{\theta }}_{N}\right)]$ is constructed to represent the steering vectors of the potential DOAs where a signal may or may not be present. An N×L matrix X is set as a zero-padded extension of S whose non-zero rows correspond to the true DOAs $\theta_{k},k=1,2,\ldots ,K$. In the presence of few stationary signal sources, X is row-sparse with K≪N. Then, the data model can be written as

(7) $$Y=\tilde{A}X+E.$$

**Figure 2:**
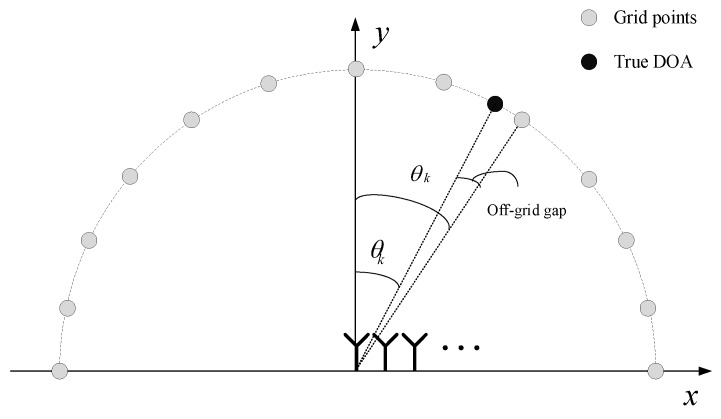
The off-grid DOA estimation model.

As the observations Y and the overcomplete matrix $\tilde{A}$ are given, our purpose is to recover the row-sparse matrix X. It is a sparse signal recovery problem with multiple measurement vectors (MMVs) in noisy circumstances. However, the DOAs are not usually exactly on the grid, which leads to the off-grid gap problem, as shown in Figure 2 [55]. In general, there are two kinds of off-grid methods to handle this problem: One is to use a fixed grid with joint estimation of the sparse signal and the grid offset [45,46]; the other one is to use a dynamic grid [18,47,48]. Compared with fixed grid methods, dynamic grid methods have the advantages of lower computational complexity and higher accuracy.

## 3. The Proposed DOA Estimation Algorithm

In this section, a robust off-grid DOA estimation algorithm is proposed to accurately estimate the azimuth angles of the target vehicles in vehicle localization. Based on the SBL formulation, all the observed and unknown variables are assumed to be stochastic and their joint prior probability distribution is specified. The joint distribution can be factored into individual prior or conditional distributions of parameters which can be estimated via a fast evidence maximization method [36]. Furthermore, an advanced grid refining strategy is designed to address the off-grid gap problem.

### 3.1. Sparse Bayesian Framework

Since the additive noise E is regarded to be complex Gaussian with noise variance $\sigma^{2}$, $p(Y|X;\sigma^{2})$ is also complex Gaussian. Thus, for each $\left\{y(t),x(t)\right\}$ pair, we have the likelihood of the array output as

(8) $$p\left(y\left(t\right)|x\left(t\right);\sigma^{2}\right)={\left(\pi \sigma^{2}\right)}^{-M}\exp(-\frac{1}{\sigma^{2}}\|y\left(t\right)-\tilde{A}x\left(t\right){\|}_{2}^{2}),$$

and hence

(9) $$p\left(Y|X;\sigma^{2}\right)=\Pi_{t=1}^{L}p\left(y\left(t\right)|x\left(t\right);\sigma^{2}\right).$$

Following the commonly used SBL model [35], the source amplitudes X are assumed to be independent in terms of both snapshots and DOAs and follow a zero-mean complex Gaussian distribution. We have

(10) $$p\left(X;\alpha \right)=\Pi_{t=1}^{L}\mathrm{CN}\left(x\left(t\right)|0,\Lambda \right),$$

where $\alpha ={\left[\alpha_{1},\alpha_{2},\ldots ,\alpha_{N}\right]}^{T}$ with $\alpha_{i}$ being the inverse variance, and Λ=diag(α). The hyperparameters α control the sparsity of the model. When $\alpha_{i}=0$, the corresponding source amplitude equals zero with probability 1. Thus, we estimate the hyperparameters α rather than the complex source amplitudes X, leading to significant reduction of parameters needed to be estimated.

Combining the likelihood of the array observations and the prior, the posterior distribution of X can be easily found by Bayes rule, and it is also a complex Gaussian with hyperparameters α and $\sigma^{2}$:

(11) $$p\left(Y|X,\alpha ,\sigma^{2}\right)=\frac{p\left(Y|X;\sigma^{2}\right)p\left(X;\alpha \right)}{p(Y;\alpha ,\sigma^{2})}=\Pi_{t=1}^{L}p\left(x\left(t\right)|\mu \left(t\right),\Sigma \right),$$

with posterior mean and covariance given by [35]

(12) $$M=\left[\mu_{1},\mu_{2},\ldots ,\mu_{L}\right]=\Lambda {\tilde{A}}^{H}\Sigma_{y}^{-1}Y,$$

(13) $$\Sigma =\Lambda -\Lambda {\tilde{A}}^{H}\Sigma_{y}^{-1}\tilde{A}\Lambda ,$$

where $\Sigma_{y}^{-1}$ denotes the inverse of the array data covariance $\Sigma_{y}$, and $\Sigma_{y}=E\left\{y\left(t\right)y{\left(t\right)}^{H}\right\}=\sigma^{2}I_{N}+A\Lambda A^{H}$.

### 3.2. Hyperparameter Estimation

The hyperparameters α and $\sigma^{2}$ are estimated by an evidence maximization procedure [56], in which the unknown source amplitudes X are treated as nuisance parameters. The evidence is the product of the likelihood (9) and the prior (10) integrated over X, which is a function of α and $\sigma^{2}$:

(14) $$p\left(Y;\alpha ,\sigma^{2}\right)=\int p\left(Y|X;\sigma^{2}\right)p\left(X;\alpha \right)dX=\frac{\exp\{-\mathrm{tr}\left(Y^{H}\Sigma_{y}^{-1}Y\right)\}}{{\left(\pi^{N}\det\Sigma_{y}\right)}^{L}},$$

and the marginal log-likelihood is

(15) $$p\left(Y;\alpha ,\sigma^{2}\right)\propto -\mathrm{tr}\left(Y^{H}\Sigma_{y}^{-1}Y\right)-L\ln\left(\det\Sigma_{y}\right).$$

The hyperparameters α and $\sigma^{2}$ are obtained by maximizing Formula (15). The expectation maximization algorithm [35] is commonly used to maximize Formula (15), which is adopted by lots of methods [17,18,45,46,47]. Recently, a fast evidence maximization method has been proposed in an on-grid DOA estimation method named MSBL [36], in which the hyperparameters are updated alternatively. Since the fast evidence maximization method is proved to be faster than the expectation maximization algorithm and its local convergence is proved by [50], we adopted it for hyperparameters estimation in this paper. Readers can refer to [36] for more derivative details that are omitted here for brevity. We have

(16) $$\alpha_{m}^{new}=\frac{\alpha_{m}^{old}}{\sqrt{L}}{\left\|Y^{H}\Sigma_{y}^{-1}a_{m}\right\|}_{2}/\sqrt{a_{m}^{H}S_{y}^{-1}a_{m}},$$

(17) $${\left(\sigma^{2}\right)}^{new}=\frac{1}{N-K}\mathrm{tr}\left(\left(I_{N}-A_{M}A_{M}^{+}\right)S_{y}\right),$$

where $S_{y}=YY^{H}/L$, $A_{M}$ is composed of active columns from A that correspond to the η largest peaks in α, and $A_{M}^{+}={\left(A_{M}^{H}A_{M}\right)}^{-1}A_{M}^{H}$. If the number of sources *K* is available, η=K; otherwise, η<M is recommended. As stated in [36], different choices of η only influence the convergence, and any choice 0<η<M provides a better estimation than the method in [35].

### 3.3. Advanced Grid Refining Strategy

As mentioned in Section 2, the assumption that all DOAs are located on the predefined grid may not be met in reality. To handle this problem, there are two kinds of methods: the fixed grid methods and the dynamic grid methods. Since the dynamic grid methods have many advantages over the fixed grid methods, we provide a dynamic grid refining strategy consisting of a root process and a step process to eliminate the off-grid gap.

#### 3.3.1. The Root Process

Considered as parameters, the grid points get refined by maximizing Formula (15). The maximization process is converted to a root seeking process [47]:

(18) $$\left[\upsilon_{{\tilde{\theta }}_{i}},1,\upsilon_{{\tilde{\theta }}_{i}}^{-1},\ldots ,\upsilon_{{\tilde{\theta }}_{i}}^{-(M-2)}\right]\left[\begin{array}{c} \frac{M(M-1)}{2}\phi^{\left(i\right)}\\ \varphi_{2}^{\left(i\right)}\\ 2\varphi_{3}^{\left(i\right)}\\ \vdots \\ \left(M-1\right)\varphi_{M}^{\left(i\right)} \end{array}\right]=0,$$

with

(19) $$\phi^{\left(i\right)}≜\sum_{t=1}^{L}\left(\right|\mu_{ti}{|}^{2}+\gamma_{ii}),$$

(20) $$\varphi^{\left(i\right)}≜L\sum_{j\neq i}\gamma_{ji}a_{j}-\sum_{t=1}^{L}\mu_{ti}^{H}y_{t-i},$$

where $\upsilon_{{\tilde{\theta }}_{i}}≜e^{-j2\pi d/\lambda \sin({\tilde{\theta }}_{i})}$, $a_{i}$,$\mu_{ti}$, and $\gamma_{ij}$ denote the *i*th column, the *i*th element, and the (i,j)th element of $\tilde{A}$, μ(t), and Σ, respectively. In addition, $y_{t-i}≜y\left(t\right)-\sum_{j\neq i}\mu_{tj}a_{j}$.

Note that Equation (18) has M−1 roots in the complex plane since its order is M−1. In practice, the closest root to the unit circle is selected (which is denoted by $z_{i^{*}}$) in the noisy environment. Then, the candidate point for grid refining is

(21) $${\tilde{\theta }}_{i^{*}}^{new}=\arcsin\left(-\frac{\lambda }{2\pi d}\mathrm{angle}\left(z_{i^{*}}\right)\right),$$

and ${\tilde{\theta }}_{i^{*}}^{new}$ will be accepted if it falls into the set of $\left[\frac{{\tilde{\theta }}_{i^{*}-1}+{\tilde{\theta }}_{i^{*}}}{2},\frac{{\tilde{\theta }}_{i^{*}}+{\tilde{\theta }}_{i^{*}+1}}{2}\right]$.

#### 3.3.2. The Step Process

The root method [47] is susceptible to the location of grid points and DOAs, especially in a coarse grid condition. If a DOA is closed to the middle of two adjacent grid points, the total consumed time will increase and the mean square error (MSE) will become the largest [57]. To address this issue, a step process is designed in our method. ${\tilde{\theta }}_{i^{*}}^{new}$ is compared with its value in the last iteration firstly, and then the grid point for grid refining is obtained as follows:

If ${\tilde{\theta }}_{i^{*}}^{new}>{\tilde{\theta }}_{i^{*}}$,

(22) $${\tilde{\theta }}_{i^{*}}^{new}={\tilde{\theta }}_{i^{*}}+\theta_{step},$$

otherwise,

(23) $${\tilde{\theta }}_{i^{*}}^{new}={\tilde{\theta }}_{i^{*}}-\theta_{step},$$

where ${\tilde{\theta }}_{i^{*}}$ is the value of ${\tilde{\theta }}_{i^{*}}^{new}$ in the last iteration, $\theta_{step}$ is a parameter with $\theta_{step}<1^{\circ }$. The influence of different vaules of $\theta_{step}$ will be discussed in the next section. Then, we discuss the stop criterion for the step process. The extreme case is that a DOA is right in the middle of two adjacent grid points. The initial grid interval is assumed to be *r*. The number of iterations may be the round of $\frac{r}{2\theta_{step}}$ for ${\tilde{\theta }}_{i^{*}}^{new}$ to get very close to the DOA under the noiseless condition. Therefore, we set the round of $\frac{r}{2\theta_{step}}$ as the maximum iteration time for the step process. When the step process reaches its stop criterion, the grid will continue to get updated via the root process until convergency.

As suggested in [47], we update active grid points rather than all grid points in each iteration. The first η maxima mean power of rows of X are regarded as the active grid points. The mean power estimation of the *i*th row of X can be calculated by $P\left(i\right)=\sqrt{\sum_{t=1}^{L}{\left|\mu_{ti}\right|}^{2}},i=1,2,\ldots ,N$, where $\mu_{ti}$ is estimated by Equation (12) in each iteration.

The stop criterion of the estimation is: $\frac{{∥\alpha^{i+1}-\alpha^{i}∥}_{2}}{{∥\alpha^{i}∥}_{2}}<\tau$ or the iteration reaches the predefined maximum time, where superscript *i* represents iteration time and τ is a settled tolerance.

**Figure 3:**
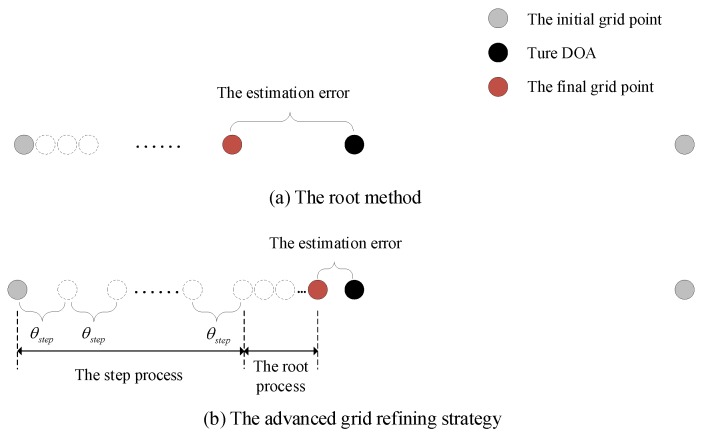
The grid refining procedures of the root method and the advanced grid refining strategy.

The intuitive illustration of the grid refining procedures of RSBL and the proposed method is given in Figure 3. Figure 3a shows the root method of RSBL, while Figure 3b exhibits the advanced grid refining strategy of the proposed method. A true DOA is assumed to be in the middle of two adjacent initial grid points. For RSBL, an initial grid point is getting closer and closer to the true DOA via the root method after each iteration until the root method is convergent. Since the distance of the grid movement via the root method during one iteration is very small, it will lead to a large error in the condition of the coarse grid with a middle located DOA and also cost much time. The proposed method can effectively address these issues. The grid refining strategy contains two processes, i.e., the step process and the root process. The step process is first conducted to greatly reduce the distance between the grid point and the true DOA and to speed up the grid refining procedure because $\theta_{step}$ is much larger than the grid movement distance via the root method. Then, the root process is implemented to further reduce the estimation error. The influence of the location of grid points and DOAs of the proposed method is therefore much less than that of RSBL. Note that, although the grid point may move in the opposite direction of the true DOA in some iterations under the bad conditions, the general trend of movement is to get closer to the true DOA.

### 3.4. Operating Instruction

The operating instruction of the proposed method can be summarized as Table 1.

Both RSBL [47] and the proposed method are dynamic grid DOA estimation methods based on SBL. However, they are different from each other. First, they employed different hyperparameters estimation methods. An expectation maximization method is used in RSBL to estimate the hyperparameters, whereas a fast evidence maximization is adopted in the proposed method, which can achieve fast convergence. Second, they implemented different grid refining strategy. RSBL uses a polynomial root method to update the grid points, whereas the proposed method provides an advanced grid refining strategy consisting of a root process and a step process. The advanced grid refining strategy accelerates the iterative convergence and is less sensitive to the location of grid points and DOAs, which makes the proposed method more effective, robust, and accurate than RSBL. The improvement of the proposed method will be verified in the next section.

## 4. Simulation

In this section, the performance of the proposed vehicle localization system is mainly evaluated by the performance of the proposed DOA estimation method. We compare the proposed method with OGSBI [45], RSBL [47], $l_{1}$-SVD [33], and MSBL [36] on accuracy and computational complexity. All the numerical simulations were carried out by MATLAB (R2016b, MathWorks, Natick, MA, USA) on a PC with an Intel i3-7350k CPU (Santa Clara, CA, USA) and 8 GB of RAM. As a representative of the on-grid model, the grid refinement strategy was not performed in $l_{1}$-SVD. OGSBI was conducted without the SVD process.

In the following simulations, a ULA composed of M=10 sensors with d=λ/2 was used to receive K=2 signals. The convergence parameters were set the same as the settings in [45]: $\rho =0.01,c=d=10^{-4}$. $\sigma^{2}$ and all the elements of α were set to be 1. The tolerance $\tau =10^{-3}$ and the maximum iteration time was 1000. It is assumed that two sources were uniformly chosen from the intervals of $\left[-20^{\circ },-10^{\circ }\right]$ and $\left[20^{\circ },30^{\circ }\right]$, respectively. The estimation errors were investigated by the MSE defined as:

(24) $$MSE=\frac{1}{K\xi }\sum_{k=1}^{K}\sum_{i=1}^{\xi }{\left({\hat{\theta }}_{i,k}-\theta_{k}\right)}^{2},$$

where ξ=200 is the total number of Monte Carlo trials and ${\hat{\theta }}_{i,k}$ is the estimated result of $\theta_{k}$ in the *i*th Monte Carlo simulation.

### 4.1. Performance Analysis

**Figure 4:**
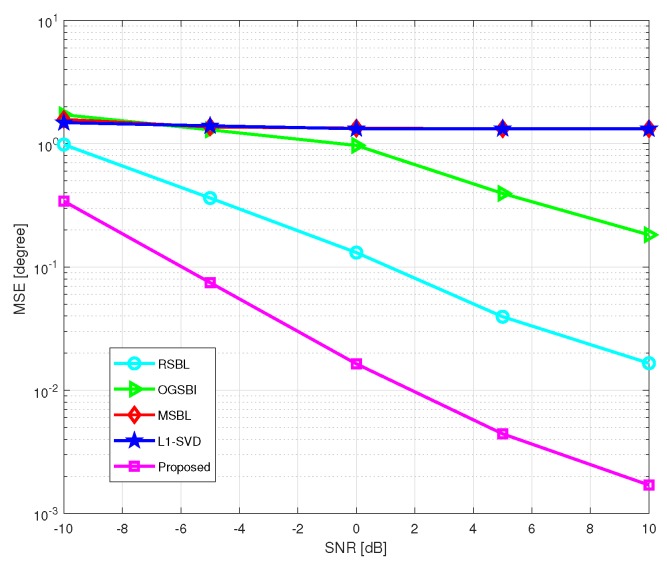
Mean square error (MSE) of DOA estimation versus signal-to-noise ratio (SNR) with $r=4^{\circ }$ and L=200.

We verify the improvement of the proposed method in terms of the MSE and the computational time. For this simulation, we set η=2 and $\theta_{step}=0.5^{\circ }$ for the proposed method, and η=2 for RSBL. Figure 4 shows the MSE of DOA estimation versus SNR, where the grid interval $r=4^{\circ }$ and the number of snapshots L=200. SNR varies from −10 dB to 10 dB. As shown in Figure 4, while SNR increases, the MSEs of $l_{1}$-SVD and MSBL almost keep constant and the MSEs of the other three methods decrease. It is clear that the proposed method has the lowest MSE among the five methods. The main reason is that $l_{1}$-SVD and MSBL are on-grid methods, whose performance in a coarse grid condition is mainly limited by the off-grid error. Therefore, the increase of SNR cannot effectively reduce this error. The other three methods are off-grid methods, which can effectively handle the off-grid gap. RSBL and the proposed method perform better than OGSBI. This is because the linear approximation used by OGSBI brings large model error in the coarse grid condition, while grid refining methods adopted by RSBL and the proposed method effectively alleviate the off-grid gap. Compared with RSBL, the proposed method can achieve higher accuracy. The performance of RSBL is seriously affected by the location of grid points and DOAs. If a DOA is right in the middle of two adjacent grid points, the performance of RSBL becomes the worst. On the contrary, if the DOA is close to a grid point, the MSE decreases nearly to a noise level. However, for the proposed method, the advanced grid refining strategy makes the refining grid points much closer to the true DOAs, especially in the case of coarse grid and middle located DOAs. Therefore, the influence of the location of grid points and DOAs of the proposed method is much less than that of RSBL.

**Figure 5:**
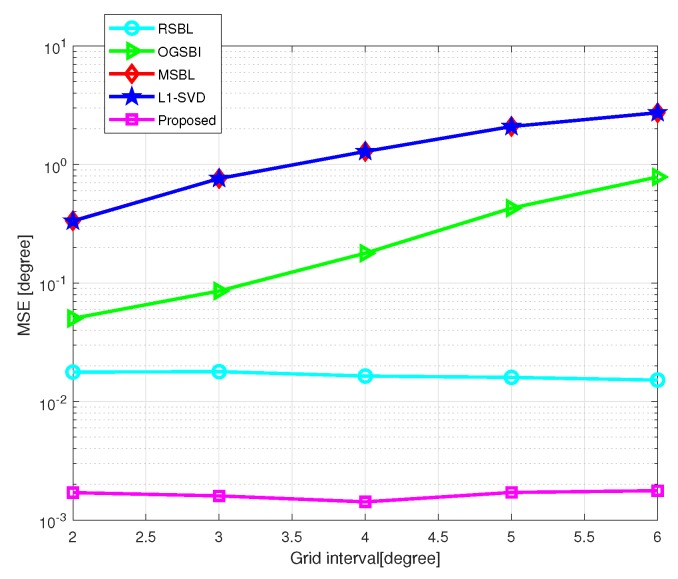
MSE of DOA estimation versus grid interval with SNR=10 dB and L=200.

Figure 5 shows the MSE of DOA estimation versus grid interval, where SNR=10 dB and the number of snapshots L=200. The grid interval varies from $2^{\circ }$ to $6^{\circ }$. As shown in Figure 5, off-grid methods outperform on-grid methods in general, and the proposed method outperforms the state-of-art methods. When the grid interval becomes larger, the MSEs of $l_{1}$-SVD, MSBL, and OGSBI increase, while RSBL and the proposed method still maintains high accuracy. This is because grid refining methods, in which the grid points are considered as adjustable parameters, adopted by RSBL and the proposed method, can effectively handle the modeling error caused by a coarse grid. The proposed method effectively eliminates the influence of the location of grid points and DOAs, which greatly affects RSBL.

**Figure 6:**
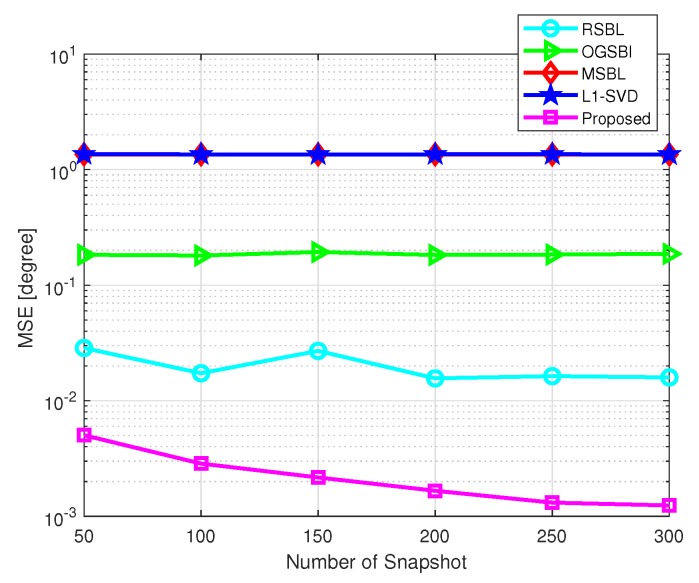
MSE of DOA estimation versus the number of snapshots with SNR=10 dB.

Figure 6 shows the MSE of DOA estimation versus the number of snapshots, where the grid interval is set as $4^{\circ }$ and SNR=10 dB. The number of snapshots varies from 50 to 300. As shown in Figure 6, the proposed method has the lowest MSE among the five methods. With the increased number of the snapshots, the MSEs of $l_{1}$-SVD, MSBL, and OGSBI almost remain unchanged, while that of RSBL and the proposed method decrease. It is because the performances of $l_{1}$-SVD, MSBL, and OGSBI in coarse grid condition are mainly limited by the model error, and the increased number of snapshots cannot effectively reduce this error. RSBL and the proposed method can effectively handle this model error via grid refining methods. Furthermore, the proposed method efficiently eliminates the influence of the location of grid points and DOAs while RSBL does not.

**Figure 7:**
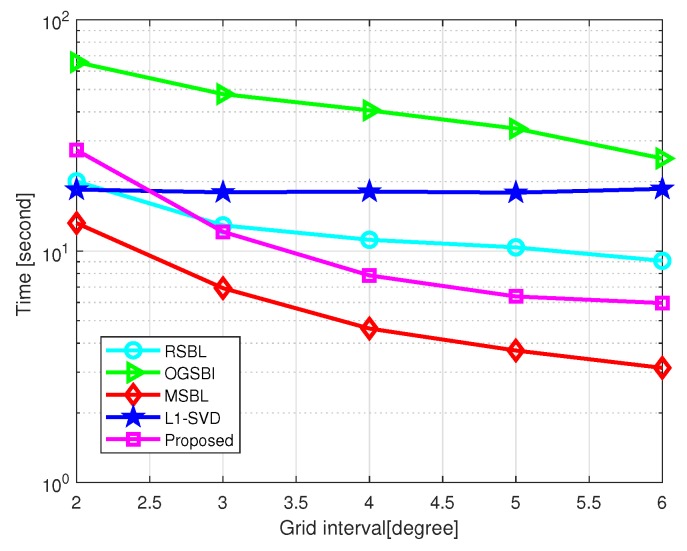
Computational time versus grid interval with SNR=10 dB and L=200.

Figure 7 shows the total CPU time of the five methods versus grid interval, where SNR=10 dB and the number of snapshots L=200. Simulation was carried out by MATLAB on a PC with an Intel i3-7350k CPU and 8 GB of RAM. The grid interval varies from $2^{\circ }$ to $6^{\circ }$. As shown in Figure 7, MSBL is the fastest of all, and the proposed method is the fastest among the off-grid methods when the grid is coarse. In general, the off-grid methods are able to address the off-grid gap at the cost of much more computational workload. When the grid interval is greater than $3^{\circ }$, RSBL and the proposed method are still more efficient than $l_{1}$-SVD, and the proposed method is faster than RSBL. For the proposed method, the advanced grid refining strategy greatly speeds up the procedure of grid refining, which makes it more efficient than RSBL.

### 4.2. Influence of Parameter Settings and Conditions

**Figure 8:**
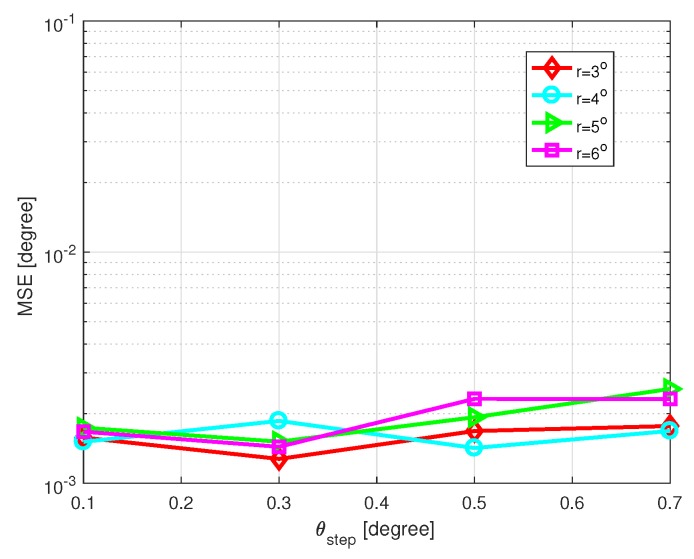
MSE of DOA estimation versus $\theta_{step}$ with SNR=10 dB and L=200.

**Figure 9:**
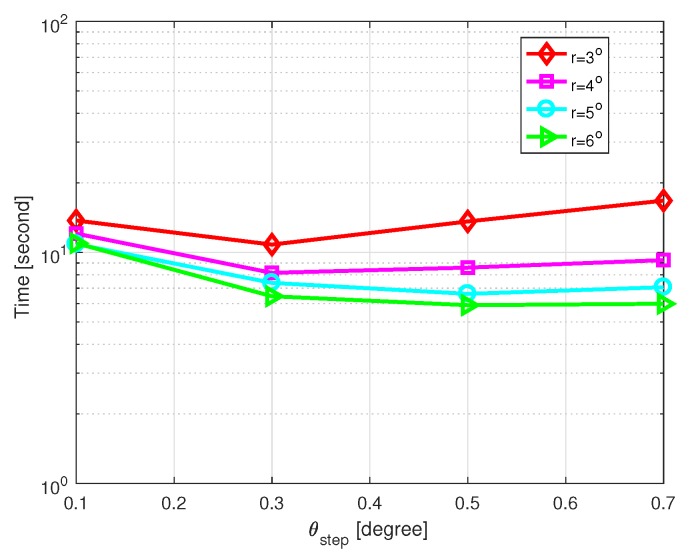
Computational time of DOA estimation versus $\theta_{step}$ with SNR=10 dB and L=200.

First, we investigate the influence of the parameter $\theta_{step}$ on the DOA estimation performance. SNR is set as 10 dB, the number of snapshots L=200 and η=2. Figure 8 shows the MSE of DOA estimation versus $\theta_{step}$ with different grid intervals, and Figure 9 shows the CPU time of DOA estimation versus $\theta_{step}$ with different grid intervals, where the grid intervals are set as $r=3^{\circ },4^{\circ },5^{\circ }$, and $6^{\circ }$, respectively. Simulations were carried out by MATLAB on a PC with an Intel i3-7350k CPU and 8 GB of RAM. In short, different values of $\theta_{step}$ do not affect the performance much. A relatively large value of $\theta_{step}$ brings light efficiency in coarse grid conditions. In general, $\theta_{step}=0.3^{\circ }$ or $0.5^{\circ }$ is recommended.

**Figure 10:**
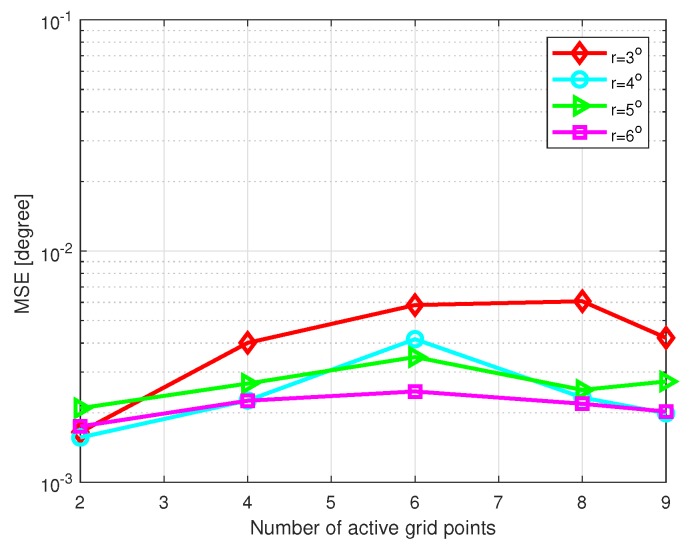
MSE of DOA estimation versus the number of active grid points with SNR=10 dB and L=200.

**Figure 11:**
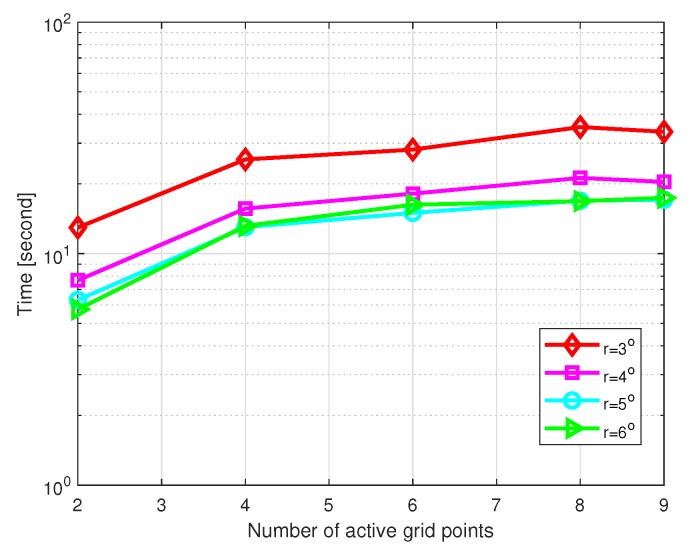
Computational time of DOA estimation versus the number of active grid points with SNR=10 dB and L=200.

Then, we investigate the influence of the number of active grid points on the DOA estimation performance. SNR is set as 10 dB, the number of snapshots L=200, and $\theta_{step}=0.5^{\circ }$. Figure 10 shows the MSE of DOA estimation versus the number of active grid points with different grid intervals, and Figure 11 shows the CPU time of DOA estimation versus the number of active grid points with different grid intervals, where the grid intervals are set as $r=3^{\circ },4^{\circ },5^{\circ }$, and $6^{\circ }$, respectively. Simulations were carried out by MATLAB on a PC with an Intel i3-7350k CPU and 8 GB of RAM. It is shown that the number of active grid points barely affects the performance in coarse grid conditions. However, if a relatively large value is chosen, there is a slight increase in computational cost.

**Figure 12:**
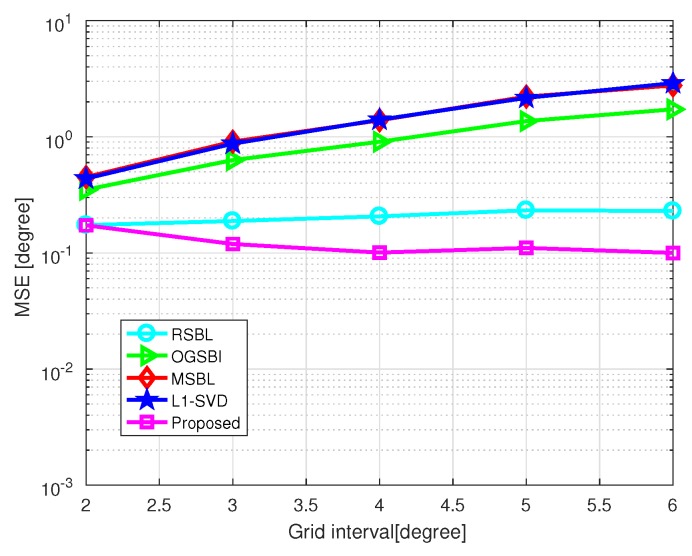
MSE of DOA estimation versus grid interval with SNR=0 dB and L=30.

Finally, we illustrate the influence of different conditions on the proposed method. Figure 12 shows the MSE of DOA estimation versus grid interval with SNR=0 dB and L=30. Compared with Figure 5, which shows the MSE versus grid interval with SNR=10 dB and L=200, the improvement of the proposed method in a bad condition becomes less significant compared with a good condition with higher SNR and a larger number of snapshots. Since the advanced grid refining strategy of the proposed method is an adaptive process, it thus will be affected by the SNR as well as the number of snapshots. Despite that, the proposed method still performs better than other methods even in bad conditions and the improvement of the proposed method will be strengthened in good conditions with a large number of snapshots and high SNR.

### 4.3. Compared with GRDOA

Both GRDOA [48] and the proposed method are off-grid DOA estimation methods based on SBL. The discrete grid points are regarded as the dynamic parameters in both methods to reduce the model error, and the performance degradation in RSBL [47] caused by middle located DOAs is effectively alleviated in both methods. However, the essential differences between GRDOA and the proposed method are:

(1) Different grid structures: the overall grid structure of GRDOA varies during the algorithm execution. Some new grid points are generated by the fission process, whereas some are discarded according to a certain criterion in the initial estimation of GRDOA. However, the overall grid structure of the proposed method stays unchanged.
(2) Different hyperparameters estimation methods: an expectation maximization method is used in GRDOA to estimate the hyperparameters, whereas a fast evidence maximization method is used in the proposed method.
(3) Different grid refining strategies: the polynomial root strategy [47] is adopted in the grid update process of GRDOA, whereas an advanced grid refining strategy consisting of a root process and a step process is provided in the proposed method.

Simulations are conducted to compare the proposed method with GRDOA and RSBL. Assume that two sources were uniformly chosen from the intervals of $\left[-20^{\circ },-10^{\circ }\right]$ and $\left[20^{\circ },30^{\circ }\right]$, respectively. $\rho =0.01,c=d=10^{-4}$. $\sigma^{2}$ and all the elements of α were set to be 1. The tolerance $\tau =10^{-3}$ and the maximum iteration time was 1000. The number of active grid points η=2. $\theta_{step}=0.5^{\circ }$ for the proposed method. Simulations are based on 200 Monte Carlo trials.

**Figure 13:**
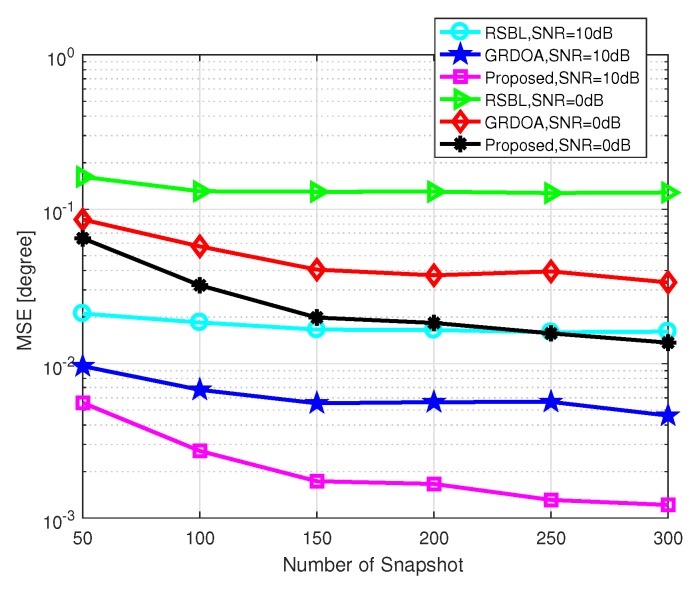
MSE of DOA estimation versus the number of snapshots with the grid interval $r=4^{\circ }$.

Figure 13 shows the MSE of DOA estimation versus the number of snapshots, where the grid interval is set as $4^{\circ }$. As shown in Figure 13, both GRDOA and the proposed method achieve higher accuracy than RSBL, since they can effectively reduce the performance degradation caused by middle located DOAs. The proposed method performs better than GRDOA. There are three main reasons. First, the fission process of GRDOA may introduce additional errors. When a new grid point is generated by the fission process, a new element corresponding to that grid point should be added to some parameters. Because the value of this new element is calculated based on some assumptions and principles, it will lead to a gap between the calculated value and the real value. Second, the performance degradation problem caused by middle located DOAs still exists in GRDOA because the root method [39] is used in the grid update process of GRDOA. In other words, even though the fission process can address this issue to some extent, the fundamental problem has not been fully solved. For the proposed method, an advanced grid refining strategy is used for grid refining which fully solves the middle located DOA problem. Finally, since the final grid number of GRDOA is quite small, additional model error may be introduced to some extent. This is because, for off-grid methods, a small number of grid points brings high efficiency, but, at the same time, it causes a large model error in general. However, the grid number of the proposed method is much larger than that of GRDOA with the same initial grid interval, so the model error of the proposed method is much less than that of GRDOA.

**Figure 14:**
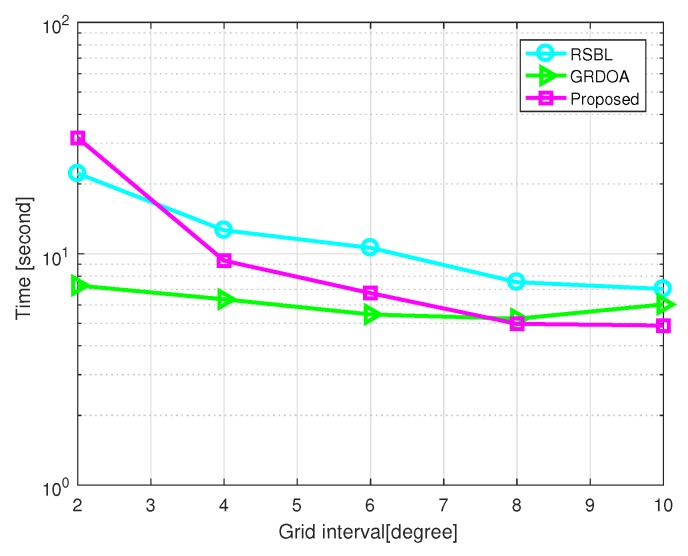
Computational time versus grid interval with SNR=10 dB and L=200.

Figure 14 shows the total CPU time versus grid interval, where SNR=10 dB and the number of snapshots L=200. Simulation was carried out by MATLAB on a PC with an Intel i3-7350k CPU and 8 GB of RAM. GRDOA is faster than the proposed method when $r<8^{\circ }$. As the grid number of GRDOA is much smaller than that of the proposed method under a relatively fine grid condition, GRDOA achieves more efficiency than the proposed method. However, this superiority of GRDOA decreases as the grid interval grows. The proposed method is faster than GRDOA when $r>8^{\circ }$. Furthermore, the proposed method shows more efficiency than RSBL when the grid interval is greater than $3^{\circ }$, as the advanced grid refining strategy greatly speeds up the procedure of grid refining.

In general, the proposed method is more suitable than GRDOA for the vehicle localization system, as the accuracy of the vehicle localization system is highly dependent on the accuracy of the DOA estimation and the proposed DOA method shows a great improvement in the accuracy.

### 4.4. Vehicle Localization

**Figure 15:**
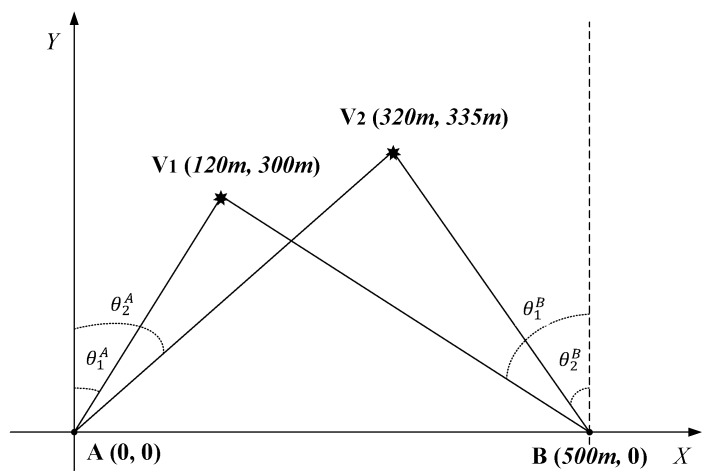
Localization diagram for two vehicles.

The vehicle localization performance of different DOA estimation methods is tested based on the proposed localization model, where the grid interval is set as $4^{\circ }$. For this simulation, we set η=2 and $\theta_{step}=0.5^{\circ }$ for the proposed method, and η=2 for RSBL. As shown in Figure 15, there are two vehicles with position coordinates $V_{1}$ (120 m, 300 m) and $V_{2}$ (320 m, 335 m), respectively. Two stations of a bistatic passive radar are located at A(0,0) and *B*(500 m, 0), respectively. Theoretically, the DOAs of these two vehicles with respect to *A* and *B* are $\theta_{1}^{A}=21.8^{\circ }$, $\theta_{1}^{B}=-51.71^{\circ }$ and $\theta_{2}^{A}=43.69^{\circ }$, $\theta_{2}^{B}=-28.25^{\circ }$. Assume that $\theta_{1}^{A*}$, $\theta_{1}^{B*}$, $\theta_{2}^{A*}$, and $\theta_{2}^{B*}$ are the estimations of $\theta_{1}^{A}$, $\theta_{1}^{B}$, $\theta_{2}^{A}$, and $\theta_{2}^{B}$, respectively, obtained by the DOA estimation algorithm. The location of $V_{1}$ can be calculated by putting $\theta_{1}^{A*}$ and $\theta_{1}^{B*}$ into (3) and (4). Similarly, the location of $V_{2}$ can be calculated by putting $\theta_{2}^{A*}$ and $\theta_{2}^{B*}$ into (3) and (4). The vehicle localization results and errors versus different DOA estimation methods with different SNR are given in Table 2, Table 3 and Table 4. By comparing these results, it can be obviously found that the localization error of the proposed method is the smallest among the four methods, which is consistent with the performance of DOA estimation.

## 5. Conclusions

In this paper, we present a passive bistatic radar-based vehicle localization system as well as a robust SBL-based off-grid DOA estimation method for vehicle localization. With this proposed DOA estimation method, a fast evidence maximization method is used to iteratively update the source powers and the noise variance. In order to address the off-grid gap issue, an advanced grid refining strategy is designed to iteratively update the discrete grid points regarded as the dynamic parameters. The position of target vehicles can be easily obtained via the DOA estimation results. Simulation results show that the proposed method exhibits superiority and effectiveness in DOA estimation as well as vehicle localization. However, the experiment was only performed by simulations since we don’t have the real experimental data yet. In the future, the verification of the proposed method with the real experimental data set is needed. In addition, hybrid vehicle localization methods, combining the proposed method with TDOA based solutions, may be a potential solution to further achieve excellent robustness and accuracy.

## Figures and Tables

**Table 1:** Operating instruction of the proposed method.

The Proposed Method.
(1) **Input**: Y;
(2) **Initialize**: α, $\sigma^{2}$, and $\theta_{step}$;
(3) **while** not converge **do**
(a) Calculate the posterior moments μ and Σ using Equations (12) and (13);
(b) Update the hyperparameters α and $\sigma^{2}$ using Equations (16) and (17);
(c) Implement the advanced grid refining strategy to update $\tilde{\theta }$:
Conduct the root process to calculate the candidate point using Equations (19)–(21);
**if** not reach the maximum iteration time of the step process
Conduct the step process using Equation (22) or Equation (23) for grid refining;
**else**
Directly use the candidate point for grid refining;
**end if**
**end while**
(4) **Output**: μ and the final grid;
(5) Achieve off-grid DOA estimation through 1D spectrum search on the final grid.

**Table 2:** Vehicle localization results and errors with SNR=−10 dB.

Method	Location $V_{1}$	Error $V_{1}$	Location $V_{2}$	Error $V_{2}$
MSBL	(126.59, 313.33)	14.87	(304.65, 338.35)	15.71
OGSBI	(126.57, 313.02)	14.58	(304.57, 338.25)	15.77
RSBL	(126.38, 309.46)	11.41	(306.34, 338.06)	14.00
Proposed	(121.81, 298.77)	2.19	(317.72, 337.13)	3.12

Multisnapshot sparse Bayesian learning (MSBL) [36], Off-grid sparse Bayesian inference (OGSBI) [45], Root sparse Bayesian learning (RSBL) [47].

**Table 3:** Vehicle localization results and errors with SNR=0 dB .

Method	Location $V_{1}$	Error $V_{1}$	Location $V_{2}$	Error $V_{2}$
MSBL	(126.59, 313.33)	14.87	(304.65, 338.35)	15.71
OGSBI	(125.77, 311.60)	12.96	(306.85, 338.51)	13.61
RSBL	(122.89, 305.56)	6.27	(314.15, 335.66)	5.89
Proposed	(119.87, 299.16)	0.85	(319.13, 333.77)	1.51

**Table 4:** Vehicle localization results and errors with SNR=10 dB .

Method	Location $V_{1}$	Error $V_{1}$	Location $V_{2}$	Error $V_{2}$
MSBL	(126.59, 313.33)	14.87	(304.65, 338.35)	15.71
OGSBI	(122.02, 304.16)	4.62	(315.04, 337.36)	5.49
RSBL	(120.81, 301.29)	1.52	(318.44, 336.06)	1.89
Proposed	(120.03, 299.84)	0.16	(320.12, 334.84)	0.20
